# Radiocarbon Dating of the Human Eye Lens Crystallines Reveal Proteins without Carbon Turnover throughout Life

**DOI:** 10.1371/journal.pone.0001529

**Published:** 2008-01-30

**Authors:** Niels Lynnerup, Henrik Kjeldsen, Steffen Heegaard, Christina Jacobsen, Jan Heinemeier

**Affiliations:** 1 Department of Forensic Medicine, University of Copenhagen, Copenhagen, Denmark; 2 AMS 14C Dating Centre, Department of Physics and Astronomy, University of Aarhus, Aarhus, Denmark; 3 Eye Pathology Section, Department of Neuroscience and Pharmacology, University of Copenhagen, Copenhagen, Denmark; Tel Aviv University, Israel

## Abstract

**Background:**

Lens crystallines are special proteins in the eye lens. Because the epithelial basement membrane (lens capsule) completely encloses the lens, desquamation of aging cells is impossible, and due to the complete absence of blood vessels or transport of metabolites in this area, there is no subsequent remodelling of these fibers, nor removal of degraded lens fibers. Human tissue ultimately derives its ^14^C content from the atmospheric carbon dioxide. The ^14^C content of the lens proteins thus reflects the atmospheric content of ^14^C when the lens crystallines were formed. Precise radiocarbon dating is made possible by comparing the ^14^C content of the lens crystallines to the so-called bomb pulse, i.e. a plot of the atmospheric ^14^C content since the Second World War, when there was a significant increase due to nuclear-bomb testing. Since the change in concentration is significant even on a yearly basis this allows very accurate dating.

**Methodology/Principal Findings:**

Our results allow us to conclude that the crystalline formation in the lens nucleus almost entirely takes place around the time of birth, with a very small, and decreasing, continuous formation throughout life. The close relationship may be further expressed as a mathematical model, which takes into account the timing of the crystalline formation.

**Conclusions/Significance:**

Such a life-long permanence of human tissue has hitherto only been described for dental enamel. In confront to dental enamel it must be held in mind that the eye lens is a soft structure, subjected to almost continuous deformation, due to lens accommodation, yet its most important constituent, the lens crystalline, is never subject to turnover or remodelling once formed. The determination of the ^14^C content of various tissues may be used to assess turnover rates and degree of substitution (for example for brain cell DNA). Potential targets may be nervous tissues in terms of senile or pre-senile degradation, as well as other highly specialised structures of the eyes. The precision with which the year of birth may be calculated points to forensic uses of this technique.

## Introduction

Living tissues undergo a continuous turnover, substitution and remodelling, both on the cytological and tissue level. In the human organism, an exception to this is the dental enamel, which after formation does not undergo any change [1]. Another highly specialised tissue is the eye lens. The transparency and refraction of the normal eye lens is dependent on the so-called crystallines. These proteins fill the cytoplasm of the highly organized eye lens fibre cells [2]. To improve optical quality, these proteins need to exist both as concentrated solutions in the outer cortical region and more glass-like in the core. The high amount of crystallines gives the lens a high refractive index which is necessary for focusing light to the retina. Lens crystallines can be classified as either classical or taxon specific [2]. The classical crystallines include members of the alpha-crystalline family and beta/gamma-crystallin superfamily [3]. An important function of these proteins is preventing protein aggregation which will make the lens opaque. Cataract occurs when the ageing crystalline proteins are no longer evenly distributed on the scale of light wavelength [4].

The lens fibre cells in the nucleus of an adult lens are thought to be produced during early embryonic life [2]. The lens plate is formed in the 4 mm embryonal stage. The lens fibre cells degrade all membrane-bound organelles and the older lens cells become compressed into the nucleus of the lens by the continuous formation of new fibres at the surface. The lens substance is thus composed of fibres that are constantly formed from elongation of the equatorial epithelium. The human lens grows rapidly in the embryo and during the first postnatal year [2]. The rate of lens growth slows between ages 1 and 10 years, and continues at a much slower, nearly linear, rate throughout life. Because the epithelial basement membrane (lens capsule) completely encloses the lens, desquamation of aging cells is impossible, and due to the complete absence of blood vessels or transport of metabolites in this area, there is no subsequent remodelling of these fibers, nor removal of degraded lens fibers [2].

**Figure 1:**
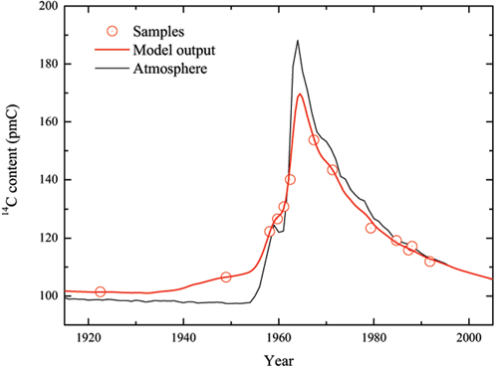
The formation of eye lens crystallines determined by ^14^C. Nuclear bomb tests during 1955–63 produced large amounts of ^14^C, which after this period has declined exponentially (thin, grey line). Comparing the amount of ^14^C in eye-lens crystallines (red circles, plotted as a function of the year of birth) with the atmospheric concentration in units of pmC (percent modern Carbon) has made it possible to investigate the timing of the formation process. The red curve shows the output from our resulting lens-formation model, which provides the basis for predicting the year of birth accurately (Fig. 2).

We have exploited the radical variations of the atmospheric ^14^C content during the last 50 years to date the formation of the lens crystallines. The concentration of ^14^C in living tissues reflects the atmospheric ^14^C content at the time of growth. This is because cosmogenic ^14^C in the atmosphere reacts with oxygen to form carbon dioxide (CO_2_), which is incorporated by plants, and then ingested by animals. The plants and animals are ultimately ingested by humans. If there is only minimal turnover in the eye lens, the content of ^14^C in the lens crystallines should thus reflect the atmospheric concentration at the time the lens crystallines were formed. The amount of ^14^C in the atmosphere (see Fig 1) was almost constant until about 1955, when nuclear-bomb tests caused it to rise dramatically [5]. Subsequent to the Test Ban Treaty in 1963 the concentration has decreased rapidly, mainly because the atmospheric ^14^C has diffused into the oceans, while radioactive decay (the half-life of ^14^C is 5730 years) is of minor importance [6]. Since the change in concentration is significant even on a yearly basis this allows very accurate dating [7].

## Results

**Figure 2:**
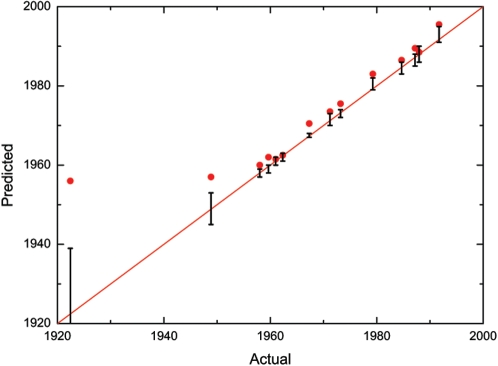
Predicting the year of birth by the ^14^C of eye lens crystallines. The year of birth of a person may be predicted from the ^14^C content of the eye-lens crystallines. In an over-simplistic picture the eye lens crystallines are formed at the birth year of the individual. The year of birth predicted in that way (red circles, showing only the centre value of the 95% confidence interval) is close to the actual yeat but evidently different, in particular for the oldest individuals. More accurate results are obtained by using our model, because it takes into account that the crystallines grow later in life too: The resulting predictions (black vertical bars, showing the 95% confidence interval) are in agreement with the actual values in all cases. The two ways of predicting the year of birth correspond to using the black (atmospheric) or the red (eye-lens model) curve in Fig. 1, respectively. The red line shows the ideal 1∶1 ratio between the predicted and actual year of birth.

The content of ^14^C in the lens crystallines was compared with the bomb pulse curve, and the intercept indicates the year of formation (Fig 1). Our results show a tight fit of less than three years between the predicted and the actual year of birth (Fig. 2). This is assuming that the lens crystallines have been formed as a single event at the year of birth, which is only a crude approximation. We therefore constructed a mathematical model based on the above assumptions on lens growth. The model was subsequently adjusted to fit the experimental data. In this way we were able to obtain detailed information concerning the timing of the formation process. The key element of our model is the formation function *F(x)* which describes the incorporation of carbon into the lens crystallines as a function of the age *x* of the individual. It is characterized by a high rate of formation ( = 1.0 in relative units) between −0.5 to 1 year after birth; an exponential decrease (half-time 0.8 years) in formation rate until the fourth year after birth; a very low linear formation rate ( = 0.027) until 25 years after birth; and finally an even lower linear formation rate ( = 0.006). The maximum lifespan is limited to 80 years. We then use the formation function to calculate the content of ^14^C (*pmC_lens_*–see Fig. 1) for a given year of birth (*b*) and age at death (*a*) on the basis of the atmospheric ^14^C concentration (*pmC_atm_*):
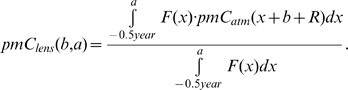
We apply a “delay” (*R*), as the source of ^14^C for the lens crystallines is ^14^C ingested with foodstuffs (either directly crop-derived, or as meat/dairy products from animals, which have fed on crops). These foodstuffs thus “delay” the atmospheric ^14^C reaching the eye lens by a growth season (set to 1 year, i.e. *R* = −1 year). For that reason we also base our ^14^C model calibration curve on biomass turnover [8] and not on the atmospheric equilibrium. Although our simple model may not describe all details of the formation process correctly, comparisons with the experimental data show that it is a good approximation and leave absolutely no possibilities for radical adjustments.

The model enables us to calculate the year of birth (Fig. 2). This was done by comparing the content of ^14^C in the lens crystallines with the plotted model curve (red curve in Fig. 1) using the ^14^C calibration program OxCal 4.0 [9], [10]. There exists good agreement between the model-predicted years of birth and the actual ones. Thus, in all cases the actual year of birth is within the predicted range. The precision of the method is demonstrated by the average magnitude of the uncertainty, which is ±1.5 year (95% confidence), excluding the case from 1922. The latter case is the oldest included in the study, and the year of birth was 30 years before onset of the bomb pulse rise. Given the extremely low formation rate of crystalline at the point when this individual lived through the bomb-pulse, this means that the ^14^C content only shows that the person was born at least 10 years before the bomb-pulse. The only other pre-pulse case, an individual born in 1948 shows increased ^14^C content, indicating that this individual, approximately 6 years old at the start of the pulse, was still forming lens crystallines.

## Discussion

In our analysis we have made the assumption that the diet of the subjects consisted mainly of terrestrial food and in particular that marine food did not contribute with a significant fraction. Consumption of fish would have a significant effect on the ^14^C content of the lenses, because the ^14^C concentration in the sea is smaller that in the atmosphere (marine ^14^C reservoir effect). However, marine food generally constitutes only a small fraction of the diet in modern Denmark, and reservoir effects are not expected to have influenced the analysis. Consumption of marine food would also have been visible in the stable-isotope ratio ^13^C/^12^C, which was also measured, but no indications of this were observed.

Our results allow us to conclude that the crystalline formation in the lens nucleus almost entirely takes place around the time of birth, with a very small, and decreasing, continuous formation throughout life. Such a life-long permanence of human tissue has hitherto only been described for dental enamel [1], [11]. In confront to dental enamel it must be held in mind that the eye lens is a soft structure, subjected to almost continuous deformation, due to lens accommodation, yet its most important constituent, the lens crystalline, is never subject to turnover or remodelling once formed. The determination of the ^14^C content of various tissues may be used to assess turnover rates and degree of substitution (for example for brain cell DNA [12]). Potential targets may be nervous tissues in terms of senile or pre-senile degradation, as well as other highly specialised structures of the eyes.

The precision with which the year of birth may be calculated points to another use of this technique. In forensics, the identification of an unidentified corpse relies on matching of data, e.g., fingerprints or DNA from the deceased with registered information. Consequently, sometimes there is a need to gather introductory data on the deceased in terms of probable sex and age. The eye lens is easily extracted, removal does not disfigure the corpse, and a result regarding the age at death of the victim may be obtained in as little as 24–48 hours.

## Materials and Methods

We obtained 13 eye lenses from 13 deceased persons of varying age. Ethical consent was obtained from the Copenhagen Board of Medical Ethics. The lenses were extracted by a minimally invasive procedure as part of autopsies performed at the Institute of Forensic Medicine, University of Copenhagen. The lens of young people is rather elastic, while the lenses of older people harden due to the accumulation of degraded by-products. It was our experience that the lens could be removed up to three days post-mortem. After this period, post-mortem degradation and putrefaction made it impossible to extract the lens, as it became more and more fluid and not intact in its capsule. After extraction, the nucleus of the lens was cut out. The nucleus of the human eye lens contains 10–15 mg tissue, corresponding to approximately 3–5 mg carbon, which is sufficient to perform a high precision accelerator mass spectrometry (AMS).
